# Molecular Basis of the Ternary Interaction between NS1 of the 1918 Influenza A Virus, PI3K, and CRK

**DOI:** 10.3390/v12030338

**Published:** 2020-03-20

**Authors:** Alyssa Dubrow, Sirong Lin, Nowlan Savage, Qingliang Shen, Jae-Hyun Cho

**Affiliations:** Department of Biochemistry and Biophysics, Texas A&M University, College Station, TX 77843, USA; alyssadubrow@tamu.edu (A.D.); showlinno1@hotmail.com (S.L.); nowlansavage@tamu.edu (N.S.); dissipative@tamu.edu (Q.S.)

**Keywords:** 1918 influenza A virus, nonstructural protein 1, CRK, PI3K, ternary interaction

## Abstract

The 1918 influenza A virus (IAV) caused the worst flu pandemic in human history. Non-structural protein 1 (NS1) is an important virulence factor of the 1918 IAV and antagonizes host antiviral immune responses. NS1 increases virulence by activating phosphoinositide 3-kinase (PI3K) via binding to the p85β subunit of PI3K. Intriguingly, unlike the NS1 of other human IAV strains, 1918 NS1 hijacks another host protein, CRK, to form a ternary complex with p85β, resulting in hyperactivation of PI3K. However, the molecular basis of the ternary interaction between 1918 NS1, CRK, and PI3K remains elusive. Here, we report the structural and thermodynamic bases of the ternary interaction. We find that the C-terminal tail (CTT) of 1918 NS1 remains highly flexible in the complex with p85β. Thus, the CTT of 1918 NS1 in the complex with PI3K can efficiently hijack CRK. Notably, our study indicates that 1918 NS1 enhances its affinity to p85β in the presence of CRK, which might result in enhanced activation of PI3K. Our results provide structural insight into how 1918 NS1 hijacks two host proteins simultaneously.

## 1. Introduction

The 1918 influenza A virus (1918 IAV) was responsible for the 1918 flu pandemic, which resulted in more than 50 million deaths worldwide [1]. Although the molecular determinant of the high virulence of 1918 IAV remains unclear, non-structural protein 1 (NS1) is considered one of the key factors in understanding the virulence of 1918 IAV. NS1 is a multifunctional virulence factor of IAVs and plays key roles in inhibiting host innate immune responses [2], such as the expression of type I interferon [3,4,5], during the infection cycle. Thus, it is considered a target for the development of anti-influenza therapeutics [6,7,8]. 

**Figure 1:**
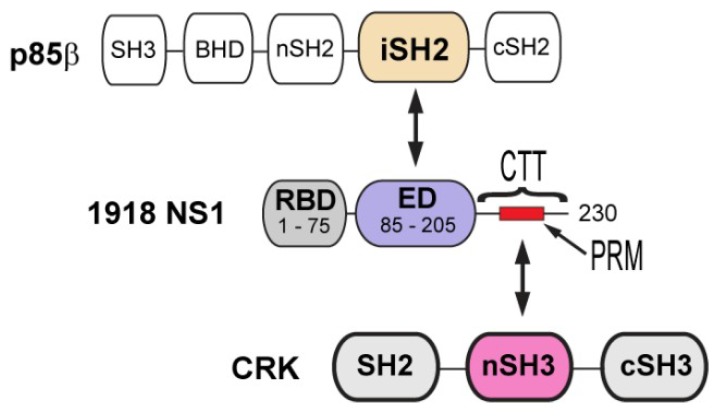
Domain organization of 1918 non-structural protein 1 (NS1), p85b, and CT-10 regulator of kinase (CRK). Arrows indicate the interacting domains between proteins.

NS1 consists of three structural units; the N-terminal RNA binding domain (RBD), an effector domain (ED), and a C-terminal tail (CTT) (Figure 1). The RBD and ED are tethered by a flexible linker, and the CTT is structurally disordered; thus, it was proposed that the conformational plasticity of NS1 is functionally important [9,10]. All three structural units are heavily involved in the interaction with a number of host proteins [9], which is the basis of the multifunctional activity of NS1. 

Phosphoinositide 3 kinase (PI3K) is one of the major binding targets of NS1. It was demonstrated that abrogating the interaction attenuated virus replication [11,12]. PI3K consists of two subunits, catalytic p110 and regulatory p85 subunits. The ED of NS1 (NS1^ED^) binds selectively to the p85β isoform; more specifically to the iSH2 domain of p85β (p85β^iSH2^) (Figure 1). Although the mechanism whereby the binding of NS1 activates PI3K remains to be determined, it was indicated that the binding interferes with the autoinhibitory interaction between p85β and the p110 catalytic subunit [13]. Subsequently, the activated p110 subunit phosphorylates Akt, resulting in the inhibition of cellular apoptosis [14,15,16] and/or affecting the cellular distribution of PI3K [17,18,19,20]. 

Recent studies have suggested that the function of NS1 varies according to influenza strains [21,22,23,24,25]; thus, it is important to examine strain-specific functions of NS1 to fully understand differential virulence among IAVs. It was demonstrated that 1918 NS1 is highly efficient at suppressing immune responses of host cells [26]. Recent studies revealed the structural bases of some distinct functions of 1918 NS1. For example, Jureka et al. showed that the RBD of 1918 NS1 directly interacts with the RIG-I CARD domain while the interaction was not observed for the protein from the Udorn strain [25]. Moreover, our laboratory revealed that the ED of 1918 NS1 (1918 NS1^ED^) binds to p85β^iSH2^ with drastically different binding characteristics to those from the ED of the Udorn strain [27]. 

Compared to other human IAV strains, 1918 NS1 contains a unique mutation in the CTT (Figure 1). While most human IAV NS1s have Thr at residue 215, it is replaced by Pro in the 1918 strain and many avian IAVs [22]. Saksela and colleagues eloquently showed that the mutation (T215P) enables 1918 NS1 to hijack CRK (CT-10 regulator of kinase) proteins during infection [22,28,29]. CRK family proteins (CRK-I, CRK-II, and CRK-L) are signaling adaptors involved in integrin-mediated signaling pathways [30]. The N-terminal SH3 (nSH3) domain of CRK recognizes a proline-rich motif (PRM) with sequence Pxx**P**xK (x = any amino acids) [31] (Figure 1); the P in bold face corresponds to P215 in 1918 NS1. 

It was shown that 1918 NS1 can co-translocate the hijacked CRK into the nucleus [28]. Although the functional outcome of the nuclear translocation of CRK remains to be revealed, it was shown that the overall tyrosine phosphorylation level of nuclear proteins increased upon CRK translocation [28]. Moreover, introducing the T215P mutation in the NS1 of the PR8 strain was shown to increase pathogenicity in a mouse model [32]. 

It was demonstrated that 1918 NS1 forms a ternary interaction with PI3K and CRK, resulting in enhanced activation of PI3K [22,29]. Therefore, it has been suggested that the unique ternary interaction between 1918 NS1, PI3K, and CRK is important for understanding the virulence of 1918 IAV [22,28,32,33]. Nevertheless, the molecular basis of this interaction remains to be determined. For example, the structural characteristics of the ternary complex has not been determined. 

Here, we characterize the binary and ternary interactions mediated by 1918 NS1 using a combination of biophysical approaches, including biolayer interferometry (BLI) and nuclear magnetic resonance (NMR). Our laboratory recently determined the crystal structure of 1918 NS1 complexed with the p85β subunit [27]. In the present study, we find that 1918 NS1 complexed with p85β hijacks CRK through a fuzzy electrostatic interaction mediated by its CTT. Our study also reveals that the ternary complex has a higher stability than those of binary complexes, providing an insight into how 1918 NS1 can achieve enhanced activation of PI3K. 

## 2. Materials and Methods 

Protein sample preparation. Genes encoding 1918 NS1 ED-CTT (residues 86–230), human p85β^iSH2^ (residues 435–599), human CRK-II (residues 1–304) and CRK-L (residues 1–303) proteins were prepared by gene-synthesis service from Genscript (Piscataway, NJ, USA). All proteins used as a ligand in BLI experiments were expressed in BL21 (DE3) *E. coli* cells (New England Biolabs Inc. Ipswitch, MA, USA) with a His_6_ and SUMO tags, and purified by Ni^2+^ NTA (nitrilotriacetic acid) column and gel-filtration chromatography. Proteins used as an analyte in BLI experiments were expressed in BL21 (DE3) *E. coli* cells with a His_6_ and SUMO tags, and purified by Ni^2+^ NTA column. The SUMO tag was removed from purified proteins by incubating the sample with SUMO protease and subsequent Ni^2+^ NTA column and gel-filtration chromatography. For the ^15^N labeled 1918 NS1^ED-CTT^, ^15^NH_4_Cl was added to M9 medium as a sole nitrogen source during protein expression. The expressed protein was purified using Ni^2+^ NTA column and gel-filtration chromatography. Purity of protein samples was confirmed using SDS-PAGE; all samples were >95% pure.

BLI experiments: the binding of 1918 NS1, p85β^iSH2^, and CRK proteins were measured at 25 °C using an Octet RED biolayer interferometer (Pall ForteBio: Fremont, CA, USA). The buffer was 20 mM sodium phosphate (pH 7.0), 100 or 1000 mM NaCl, 1% BSA, and 1mM DTT. His_6_ tagged proteins (5 µg/mL) were immobilized on Ni-NTA biosensor tips. To measure the binding affinity between CRK and the NS1:p85 complex, mixtures of 0.02–2 µM of NS1 and 15 µM p85β were used. Under this condition, > 98% of NS1 exists as a binary complex. All measurements were performed at least three times. To determine K_D_ values, five final signals in the association phase were averaged. All reported K_D_ values were determined by global fitting of three repeated results using a 1:1 binding model using Prism 8.

Nuclear magnetic resonance (NMR) assignment: NMR heteronuclear ^1^H-^15^N NOE (nuclear Overhauser effect) experiment was conducted using a protein sample in 20 mM sodium phosphate (pH 7.0), 80 mM NaCl, 0.02% sodium azide, 1 mM EDTA, and 10% D_2_O at 25 °C. NMR spectra were acquired on Bruker AVANCE 14.1 T spectrometers (Bruker BioSpin, Billerica, MA, USA), equipped with a cryogenic probe (Texas A&M Biomolecular NMR facility). A recycle delay of 10 s was used in the reference experiment. A longer recycle delay of 15 s was used in the reference experiment. The saturation of proton during steady-state was performed by applying 180° pulses for 4 s [34]. Errors of the relaxation parameters were estimated using spectrum noise level. NMR spectra were processed with NMRPipe [35] and analyzed with NMRViewJ (One Moon Scientific, Inc.). 

## 3. Results and Discussion

### 3.1. The C-Terminal Tail (CTT) of 1918 Non-Structural Protein 1 (NS1) Directly Binds to CT-10 Regulator of Kinase (CRK)

**Figure 2:**
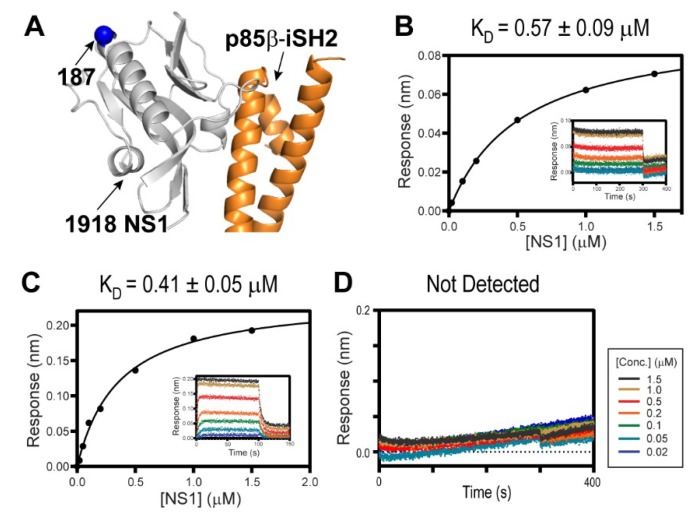
Interaction between 1918 NS1 and p85β (PDB ID: 6U28). (**A**) Crystal structure of the 1918 NS1^ED-CTT^:p85β^iSH2^ complex. The position of residue 187 is shown as a blue sphere. Biolayer interferometry (BLI)-derived binding isotherms of (**B**) 1918 NS1E^D-CTT^ and CRK-II, (**C**) 1918 NS1^ED-CTT^ and CRK-L. Insets: representative BLI sensorgrams with different analyte concentrations are shown by different colors. K_D_ values and uncertainties are the global fitting result of three repeated data. (**D**) BLI sensorgram of 1918 NS1^ED-ΔCTT^ binding to CRK-L.

To study the interaction of 1918 NS1 with PI3K and CRK, we employed full-length CRK proteins (CRK-II and CRK-L), the iSH2 domain of p85β subunit of PI3K (p85β^iSH2^), and 1918 NS1 containing ED and CTT (1918 NS1^ED-CTT^) (Figure 1). Previous studies indicated that the interaction with PI3K is mediated by monomeric form of NS1^ED^ [11,13,27]. The isolated NS1^ED^ forms a W187-mediated homodimer [36,37,38]. Although the homodimerization is weak (K_D_ ~ 89 µM) [37], it can interfere with the quantitative measurement of the characteristics of the binding between NS1 and host proteins. Thus, we incorporated an W187R substitution, which was shown to prevent homodimerization and precipitation of NS1^ED-CTT^ [37]. It should be noted that W187 is located on the opposite side of the p85β-binding site (Figure 2A). Moreover, it was shown that W187R substitution in 1918 NS1 did not affect binding to p85β^iSH2^ [27]. 

Despite the importance of the NS1:CRK interaction in understanding the virulence of the 1918 IAV [22,28,29,33], their intrinsic binding characteristics were not determined quantitatively. Using BLI, we measured the binding affinity of 1918 NS1^ED-CTT^ and full-length CRK proteins. Intriguingly, despite the difference in the intramolecular interdomain interactions between CRK-II and CRK-L, 1918 NS1^ED-CTT^ binds to CRK-L and CRK-II with a similar affinity; K_D_ = 570 nM and 410 nM, respectively (Figure 2B,C). This result is consistent with previous results using co-precipitation of transfected 1918 NS1 [22]. 

In contrast, truncating the CTT (1918 NS1^ED-ΔCTT^) abolished binding to CRK (Figure 2D), indicating that the 1918 NS1:CRK interaction is mainly mediated by the PRM in the CTT. The isolated PRM peptide is considered to be structurally disordered and lacks higher order structures [31,39]. However, the conformation of the CTT in 1918 NS1 was not directly characterized; it thus remains uncertain whether the PRM region is indeed structurally disordered. 

### 3.2. The CTT of 1918 NS1 is Structurally Flexible

Although our binding data suggested that the PRM might be exposed to solvent in the context of 1918 NS1^ED-CTT^, it is not direct evidence of high conformational flexibility of the PRM and overall CTT in the protein. We showed previously that the conformational flexibility of the PRM^1918NS1^ peptide plays a critical role in increasing its affinity to the nSH3 domain of CRK [31,39]. Thus, to understand the binding mechanism between 1918 NS1 and CRK, the conformational flexibility of the CTT within 1918 NS1^ED-CTT^ should be examined.

**Figure 3:**
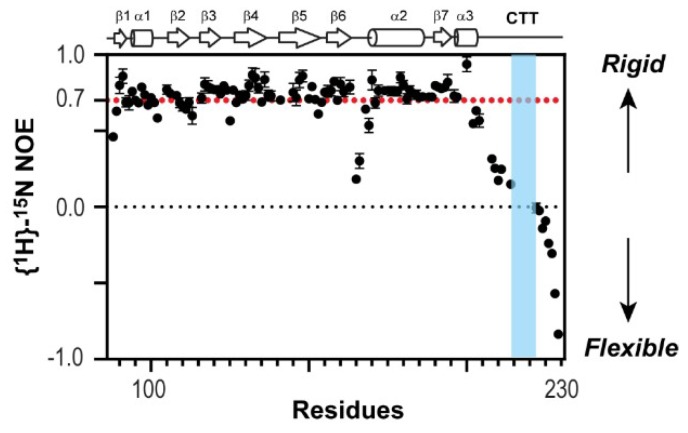
Nuclear magnetic resonance (NMR) {^1^H}-^15^N heteronuclear Overhauser effect (NOE) of 1918 NS1^ED-CTT^. Proline-rich motif (PRM) sequence is shown in blue.

To test directly whether the CTT is structurally flexible in the context of 1918 NS1^ED-CTT^, we used NMR ^1^H-^15^N heteronuclear Overhauser effect (het-NOE) [40], which is extremely sensitive to the motion of the protein backbone. Briefly, a het-NOE value below 0.7 indicates that the residue is structurally dynamic, while values ranging from 0.7 to 1.0 indicate that the conformation of the residue is rigid. We found that het-NOE values across the CTT were significantly lower than 0.7, indicating its high conformational flexibility in the protein (Figure 3). Moreover, the resonances of 10 residues belonging to the PRM (residues 212–221, except residue 214) were missing in the ^1^H-^15^N HSQC (heteronuclear single quantum coherence) spectrum owing to a line-broadening effect (blue region in Figure 3), indicating that the region undergoes conformational exchange in an intermediate NMR timescale, typically in microsecond to millisecond timescales [41,42]. Taken together, these results provide direct evidence of the highly dynamic conformation of the CTT including the PRM.

### 3.3. 1918 NS1 Forms a Ternary Complex with p85 and CRK

**Figure 4:**
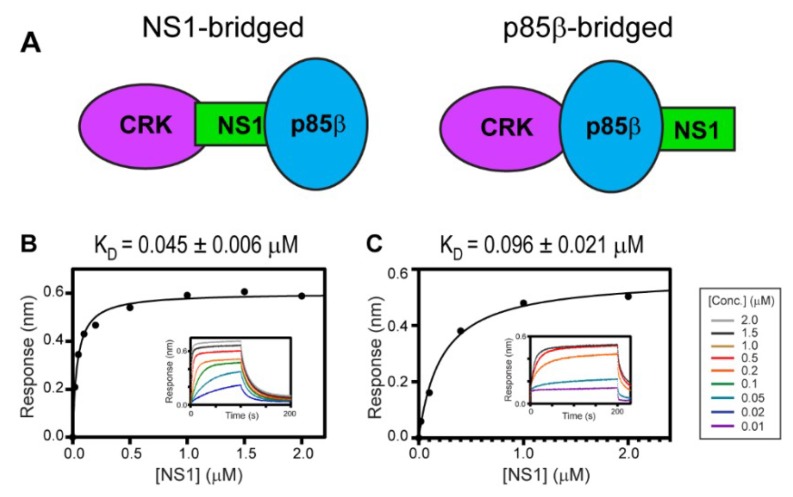
Ternary interaction between 1918 NS1, p85β, and CRK. (**A**) Schematic showing the NS1-bridged (left) and p85β-bridged (right) ternary complex. BLI-derived binding isotherms between the 1918 NS1^ED-CTT^:p85β complex and (**B**) CRK-II and (**C**) CRK-L. Insets: representative BLI sensorgrams with different analyte concentrations are shown by different colors. K_D_ values and uncertainties are the global fitting result of three repeated data.

Ylösmäki et al. showed that avian NS1, with the PRM sequence similar to 1918 NS1, forms a ternary complex with PI3K and CRK [29]. They also proposed two alternative modes of the ternary complex: NS1-bridged and p85β-bridged modes (Figure 4A). The critical distinction between the two binding modes is based on whether CRK binds to the CTT (i.e., PRM) of NS1 or to p85β.

To test whether 1918 NS1 forms a ternary complex with PI3K and CRK, we measured the binding affinity between CRK-II and 1918 NS1^ED-CTT^ complexed with p85β^iSH2^ (i.e., 1918 NS1^ED-CTT^:p85β^iSH2^ complex) (Figure 4B); K_D_ = 45 ± 6 nM. A similar binding affinity was measured between CRK-L and the 1918 NS1^ED-CTT^:p85β^iSH2^ complex; K_D_ = 96 ± 21 nM (Figure 4C). These results are direct evidence that 1918 NS1 can form a ternary complex with CRK and PI3K through the so-called NS1-bridged ternary complex.

**Figure 5:**
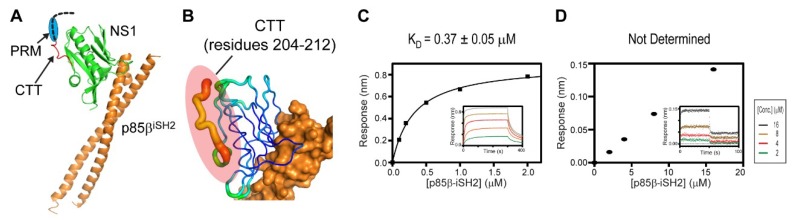
The C-terminal tail (CTT) of 1918 NS1 mediates the interaction with CRK. (**A**) Crystal structure of the 1918 NS1^ED-CTT^:p85β^iSH2^ complex. The region with missing electron densities are shown as a dashed black line. The PRM region is marked by a blue circle. (**B**) Representation of crystallographic B-factors of 1918 NS1^ED-CTT^ in complex with p85β. BLI-derived binding isotherms between p85β^iSH2^ and (**C**) 1918 NS1^ED-ΔCTT^ and (**D**) CRK-II. The result of p85β^iSH2^ and CRK-II was not fit because the affinity was too weak to fit reliably. Insets: representative BLI sensorgrams with different analyte concentrations shown by different colors. K_D_ values and uncertainties are the global fitting result of three repeated data.

To further understand the structural basis of the ternary interaction, we examined the crystal structure of the 1918 NS1^ED-CTT^:p85β^iSH2^ complex, which was recently determined by our research group (PDB ID: 6U28) [27]. Although the 1918 NS1 in the crystal structure contained the full-length CTT (residues 204–230), the electron densities of residues 213–230 in the CTT were missing in the crystal structure (Figure 5A); the missing region also included the PRM (residues 211–221). This indicates that the CTT remains highly flexible in the complex. Moreover, although the N-terminal residues (204–212) in the CTT were visible in the crystal structure, the B-factors of the region were significantly elevated compared to other regions in NS1 (Figure 5B).

These structural data suggest that most of the CTT does not interact with ED and p85β in the binary complex. To test the structural model, we measured the binding affinity between the CTT-truncated 1918 NS1 (1918 NS1^ED-ΔCTT^) and p85β^iSH2^ (Figure 5C). If the CTT interacts with p85β^iSH2^, 1918 NS1^ED-ΔCTT^ would have a lower affinity to p85β than 1918 NS1^ED-CTT^. However, we found that the K_D_ (0.37 µM) was similar to that of the 1918 NS1^ED-CTT^:p85β^iSH2^ complex (0.30 µM) [27], indicating that the CTT does not interact with p85β^iSH2^ in the complex.

Interestingly, we noticed that the affinity of CRK to the 1918 NS1:p85β^iSH2^ complex is approximately 10-fold higher than to free 1918 NS1 (Figure 2B and Figure 4B), suggesting the interaction between CRK and p85β^iSH2^. The direct CRK:p85β^iSH2^ interaction was not reported. Although it was shown that CRK binds to the PRM of p85β [43,44,45], the region is not present in p85β^iSH2^. Thus, we further tested the direct binary interaction between CRK-II and p85β^iSH2^ in the absence of 1918 NS1. Indeed, we found that CRK can interact with p85β^iSH2^ in the absence of 1918 NS1, although the interaction was too weak to be measured quantitatively (Figure 5D). To our knowledge, this is the first observation of the direct interaction between CRK and p85β^iSH2^. These data suggest that the ternary complex is stabilized by at least three disparate intermolecular interactions: 1918 NS1 CTT-CRK, 1918 NS1^ED^-p85β^iSH2^, and CRK-p85β^iSH2^ interactions. Considering the weak affinity, we expect that the direct CRK:p85β^iSH2^ interaction might be effective only via 1918 NS1. This also indicates that 1918 NS1 binds more tightly to p85β in the presence of CRK. Moreover, our structural and BLI data indicate that 1918 NS1 is able to form the ternary complex in any order, that is, 1918 NS1 binds to either CRK or PI3K and then to the rest.

### 3.4. Molecular Basis of the High Affinity of 1918 NS1:p85 Complex and CRK

Compared to affinities of CRK with its cellular binding partners (typically 1–10 µM) [46,47], the binding affinity between the 1918 NS1:p85β complex and CRK is considerably high, which might be useful for hijacking CRK proteins even in presence of natural binding partners of CRK proteins during the infection cycle. What is then the molecular basis underlying the binding affinity between CRK and the 1918 NS1:p85 complex? Although the structure of the 1918 NS1:p85β complex indicated that the CTT remained flexible, the driving force of the high affinity should be addressed.

**Figure 6:**
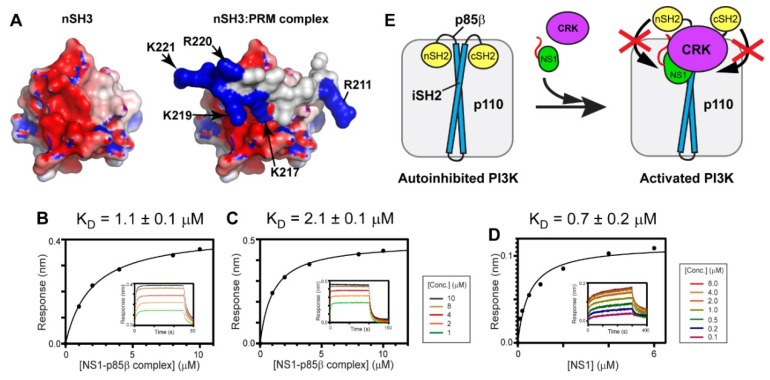
Fuzzy electrostatic interaction between CRK and the 1918 NS1:p85β complex. (**A**) Crystal structure of the free nSH3 (left) of CRK-II and its complex (right) with PRM of 1918 NS1 (PDB ID: 5UL6). The protein surface is colored according to electric potential at neutral pH from -5 kT (red) to +5 kT (blue). (right panel) Positively charged residues of PRM are shown in blue. BLI-derived binding isotherms between the 1918 NS1^ED-CTT^:p85β^iSH2^ complex and (**B**) CRK-II and (**C**) CRK-L in the presence of 1M NaCl. (**D**) BLI-derived binding isotherm between 1918 NS1^ED-CTT^ and p85β^iSH2^ in the presence of 1M NaCl. Insets: representative BLI sensorgrams with different analyte concentrations are shown by different colors. (**E**) A schematic model showing how 1918 NS1 might enhance activation of PI3K using hijacked CRK.

Intriguingly, it was shown that electrostatic interaction plays an important role in binding affinity and selectivity between nSH3^CRK^ and its cellular binding partners. For example, Knudsen et al. found that positively charged residues in the C-terminal region of cellular PRMs are critical for binding to nSH3^CRK^ [47]. Moreover, Wu et al. identified that the electron density of the C-terminal region of a PRM peptide is missing in the crystal structure of its complex with nSH3^CRK^ [48], indicating high conformational flexibility of the region. In line with these findings, our previous studies indicated that a fuzzy electrostatic interaction between the structurally dynamic PRM^1918^ peptide and the isolated nSH3^CRK^ domain drives high affinity binding [31,39]. The fuzzy electrostatic interaction was originally introduced to explain the phosphorylation-dependent ultrasensitive binding of intrinsically disordered proteins to their receptors [49]. The high conformational flexibility of PRM enables its positively charged residues to form the fuzzy electrostatic interaction with the negatively charged surface of the nSH3^CRK^ domain (Figure 6A). It was also demonstrated that the binding affinity of a PRM peptide and nSH3^CRK^ correlates with the increasing net charge of PRM, which is consistent with the theoretical model of fuzzy electrostatic interaction [31,49]. However, it remains to be tested whether the short PRM in the background of the 1918 NS1:p85β^iSH2^ complex (~36 kDa) still mediates the fuzzy electrostatic interaction with the nSH3 domain embedded in full-length CRK (34 kDa).

To test the hypothesis, we measured the K_D_ values between CRK-II and the 1918 NS1^ED-CTT^:p85β^iSH2^ complex in the presence of 1M NaCl. The fuzzy electrostatic interaction, in essence, is a long-range electrostatic interaction mediated by a conformationally flexible ligand [49]; thus, the interaction is screened in a high ionic strength solution [39]. Indeed, we observed that the K_D_ value increased by more than 24-fold in the presence of 1M NaCl (Figure 6B), compared to that in 100 mM NaCl (Figure 4B). The binding affinity between CRK-L and the 1918 NS1^ED-CTT^:p85β^iSH2^ complex was also reduced by a similar magnitude in the presence of 1M NaCl (Figure 6C and Figure 4C).

We further tested whether the affinity between 1918 NS1 and p85β^iSH2^ was affected by the presence of 1M NaCl; if the affinity of the 1918 NS1:p85β interaction is reduced in 1M NaCl, it would also reduce the stability of the ternary complex. However, the K_D_ value of the 1918 NS1:p85β complex was measured to be 0.7 µM (Figure 6D) which is close to the value in 100 mM NaCl [27]. This result is consistent with our previous finding that the interaction between 1918 NS1 and p85β^iSH2^ is mainly mediated by the hydrophobic force [27]. Taken together, we conclude that the large increase in K_D_ between CRK and the 1918 NS1^ED-CTT^:p85β^iSH2^ complex is owing to the weakened electrostatic interaction between the CTT and CRK by the high ionic strength.

Overall, our results support the idea that the fuzzy electrostatic interaction is a major driving force of high-affinity binding between CRK and the 1918 NS1:p85β complex. Although fuzzy electrostatic interactions have been implicated in the binding of other viral-host protein interactions [50], to our knowledge, this is the first report on the role of the fuzzy electrostatic interaction mediated by a viral protein in forming a multimeric protein complex.

A previous study proposed that NS1 bound to p85β^iSH2^ displaces the SH2 domains of p85β, thereby preventing the autoinhibitory function of the SH2 domains [13]. Our study showed that the ternary complex has a higher stability than the binary complex of 1918 NS1 and p85β. Taken together, we speculate that 1918 NS1, using hijacked CRK, can effectively interfere with the re-binding of the displaced SH2 domains to PI3K (Figure 6E). Further structural studies on the ternary complex will enhance our understanding at the molecular level of how 1918 NS1 enhances activation of PI3K.
